# NQO1-Dependent Redox Cycling of Idebenone: Effects on Cellular Redox Potential and Energy Levels

**DOI:** 10.1371/journal.pone.0017963

**Published:** 2011-03-31

**Authors:** Roman H. Haefeli, Michael Erb, Anja C. Gemperli, Dimitri Robay, Isabelle Courdier Fruh, Corinne Anklin, Robert Dallmann, Nuri Gueven

**Affiliations:** 1 Santhera Pharmaceuticals, Liestal, Switzerland; 2 Biozentrum, University of Basel, Basel, Switzerland; 3 Institut Straumann AG, Basel, Switzerland; 4 Novartis Pharma AG, Basel, Switzerland; 5 Institute of Pharmacology and Toxicology, University of Zürich, Zürich, Switzerland; National Institute for Medical Research, Medical Research Council, United Kingdom

## Abstract

Short-chain quinones are described as potent antioxidants and in the case of idebenone have already been under clinical investigation for the treatment of neuromuscular disorders. Due to their analogy to coenzyme Q_10_ (CoQ_10_), a long-chain quinone, they are widely regarded as a substitute for CoQ_10_. However, apart from their antioxidant function, this provides no clear rationale for their use in disorders with normal CoQ_10_ levels. Using recombinant NAD(P)H:quinone oxidoreductase (NQO) enzymes, we observed that contrary to CoQ_10_ short-chain quinones such as idebenone are good substrates for both NQO1 and NQO2. Furthermore, the reduction of short-chain quinones by NQOs enabled an antimycin A-sensitive transfer of electrons from cytosolic NAD(P)H to the mitochondrial respiratory chain in both human hepatoma cells (HepG2) and freshly isolated mouse hepatocytes. Consistent with the substrate selectivity of NQOs, both idebenone and CoQ_1_, but not CoQ_10_, partially restored cellular ATP levels under conditions of impaired complex I function. The observed cytosolic-mitochondrial shuttling of idebenone and CoQ_1_ was also associated with reduced lactate production by cybrid cells from *mitochondrial encephalomyopathy, lactic acidosis and stroke-like episodes* (MELAS) patients. Thus, the observed activities separate the effectiveness of short-chain quinones from the related long-chain CoQ_10_ and provide the rationale for the use of short-chain quinones such as idebenone for the treatment of mitochondrial disorders.

## Introduction

Quinones, such as vitamin K or coenzyme Q_10_ (CoQ_10_), are a chemical class containing a quinoid ring system [reviewed by 1,2] as pharmacophore. Despite significant differences between quinones, the quinoid system is the dominant feature that causes all of them to be electrophiles, oxidants and colored. However, already minor variances in their chemical and physicochemical properties lead to extensive differences in their biological and pharmacological effects. Enzymes involved in cellular quinone metabolism catalyze mainly two different redox reactions. For example, NADPH:cytochrome P450 reductase can generate semiquinones by incomplete, one-electron reduction [1], [2]. Since semiquinones can react with molecular oxygen to generate reactive oxygen species (ROS), this process can lead to oxidative damage of cellular macromolecules, toxicity and mutagenicity [1], [2]. In contrast, NAD(P)H:quinone oxidoreductases (NQOs) are cytosolic flavoproteins that compete with P450 reductase and catalyze the reduction of highly reactive quinones and their derivates by complete, two-electron reduction [2]. This results in the formation of relatively stable hydroquinones, often also referred to as quinols, and therefore avoids the formation of ROS. Thus, NQOs are considered key detoxifying enzymes which are induced by stressors such as xenobiotics or oxidants [3]. Currently, NQO1 and NQO2, with substantial differences in substrate specificity and expression patterns, are described. While NQO1 uses nicotinamide adenine dinucleotide (phosphate) (NADH or NADPH) as electron donor, NQO2 shows a high preference for dihydronicotinamide riboside (NRH) [3].

NQOs have been shown to reduce numerous pharmacologically active compounds such as quinone epoxides, aromatic nitro and nitroso compounds, azo dyes and Cr(VI) compounds [4]. Notably, NQO1 has its highest affinity towards quinones; for example, β-lapachone and mitomycin C exhibit their biological activity not until their NQO1-dependent bioreduction [5], [6]. Both NQO1 and NQO2 are able to reduce CoQ_0_ [7] and CoQ_1_ [8], [9]. These quinones are short-chain analogs of CoQ_10_, which is best known for its pivotal role in mitochondrial oxidative phosphorylation, although the functional significance of NQO-dependent reduction of CoQ_0_ and CoQ_1_ is still unclear.

Idebenone, a benzoquinone carrying exactly the same quinone moiety as CoQ_0_, CoQ_1_ and CoQ_10_, shows multiple activities *in vitro* and *in vivo*. Most prominently associated with idebenone is its potent antioxidant capacity as substantiated by the ability to prevent lipid peroxidation and ROS in multiple systems [10]–[14]. Consistent with this role, idebenone proved cytoprotective after cellular exposure to various toxic insults [10], [12], [13], [15]. Consequently, it is under investigation as a possible treatment for disorders characterized by excessive oxidative damage due to mitochondrial defects. Idebenone is quickly absorbed and is well tolerated and safe given as single or repeated daily doses [16]. Successful treatment of a patient with Leigh syndrome using idebenone, where high-dose CoQ_10_ had no effect on respiratory function, is indicative of significant levels of idebenone in the brain [17]. Thus, idebenone has been suggested for treating patients with *mitochondrial encephalopathy, lactic acidosis and stroke-like episodes* (MELAS) [18], [19]. Idebenone has been most intensely studied for the treatment of Friedreich's Ataxia (FRDA) [20], [21], which is a mitochondrial disorder characterized by increased sensitivity to free radicals [22]. FRDA patients also show deficient activity of mitochondrial respiratory complexes I, II and III and aconitase.

In addition to its antioxidant function, multiple activities have been reported for idebenone such as blocking of Ca^2+^-channels [23], increased synthesis of NGF [24], stimulation of mitochondrial glycerol-phosphate shuttle [25], modulation of arachidonic acid metabolism [26] and increased mitochondrial function under low oxygen [27]. Due to its structural analogy to CoQ_10_, idebenone was anticipated to participate in electron transport through the respiratory chain [11]. Indeed, idebenone interacts with mitochondrial complexes I, II and III [28], [29]. But whereas it is a good substrate for the latter two, it inhibits both the proton pumping and redox activity of mitochondrial complex I [11], [25], [29]–[31]. To what extent this activity is responsible for the beneficial effects of idebenone is still under investigation.

Here, we describe that idebenone is a substrate for reduction by NQO1 and NQO2. The NQO1-reduced idebenone is able to donate electrons into the mitochondrial respiratory chain and it can partially restore cellular adenosine triphosphate (ATP) levels under conditions of impaired complex I function. Consistent with this cytoplasmic-mitochondrial redox cycling hypothesis, idebenone also reduces lactate production in a cell culture model of MELAS. We also show that this effect is specific to some short-chain quinones such as idebenone and is not shared with the structurally related long chain quinones such as CoQ_10_.

## Results

### Reduction of short-chain quinones by NQO enzymes *in vitro*

**Figure 1:**
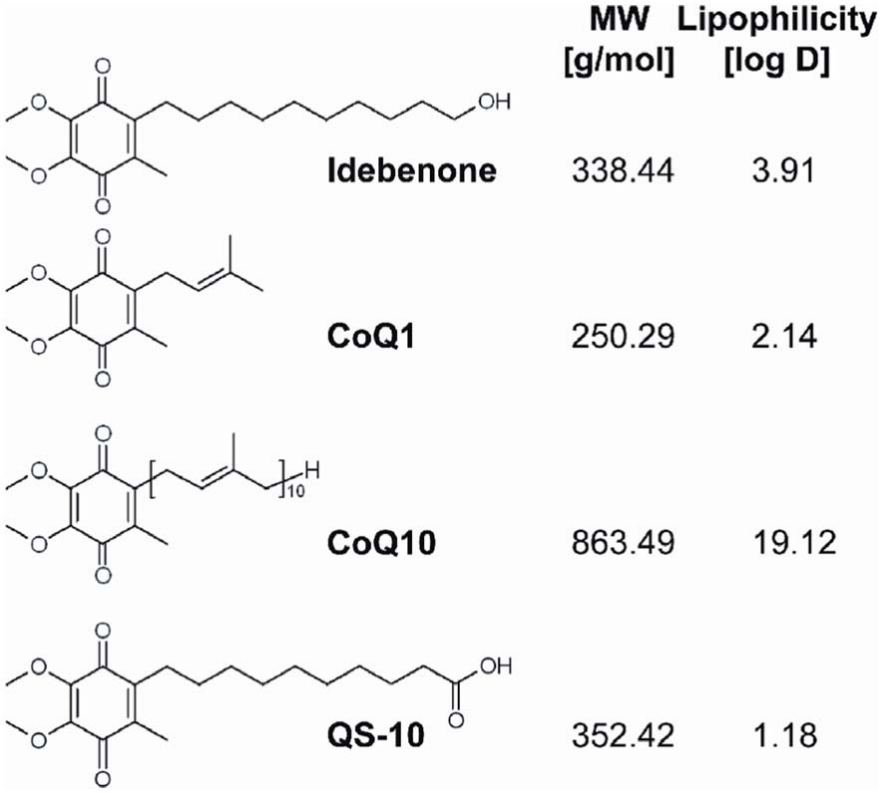
Chemical structures of the quinones tested. Idebenone, CoQ_1_, CoQ_10_ and QS-10 share the same substitution pattern of the quinone moiety but differ in the alkyl tail attached to the C6-carbon atom of their quinone ring. Whereas idebenone and QS-10 possess an alkyl chain with a terminal polar group (hydroxyl or carboxylic acid group), CoQ_1_ and CoQ_10_ contain one or ten isoprenoid repeats, respectively. Molecular weight and calculated log D value (Advanced Chemistry Development Software Package, Version 12, ACD Labs, Toronto, Canada) for each molecule are shown. Log D values are a measure for lipophilicity incorporating ionization of the compound in which small values indicate affinity for the aqueous phase.

Since NQO1 is thought to be the main cellular enzyme responsible for quinone metabolism, we were interested if this also applied to idebenone (Ide) and related quinones such as CoQ_1_ and CoQ_10_, since they share the identical substitution pattern of the quinone moiety (Figure 1). We also analyzed QS-10 (6-(9-carboxynonyl)-2,3-dimethoxy-5-methyl-1,4-benzoquinone), one of the first idebenone metabolites during oxidative side chain shortening [32]. Experiments with recombinant enzymes clearly demonstrate that these four quinones are differentially reduced by NQO1 (Table 1, Figure S1A+B). Generally, NQO1 demonstrated a slight preference of NADPH over NADH as electron donor with either idebenone, CoQ_1_ or QS-10 as acceptor substrate (Table 1). Whereas maximal reduction velocity (v_max_) for NQO1 presented in the following order: CoQ_1_ > idebenone > QS-10, we could not find any evidence for a NQO1-mediated reduction of CoQ_10_ (Table 1). Due to poor solubility of CoQ_10_ in aqueous solutions, we repeated the assay with different formulations of CoQ_10_ in accordance to its lipophilic requirements. Nevertheless, when complexed with fetal bovine serum (FBS) or incorporated into phosphatidylcholine-based liposomes [33], [34], we were unable to detect any NQO1-dependent reduction of CoQ_10_ (Figure S1C). In contrast, idebenone was clearly reduced by NQO1 under all conditions tested.

**Table 1:** Steady-state kinetic constants of NQO1 and NQO2 with different quinones.

Enzyme	NQO1				NQO2	
Substrate	NADH		NADPH		NRH-derivate*	
	K_m_ [µM]	v_max_ [µmol/mg/min]	K_m_ [µM]	v_max_ [µmol/mg/min]	K_m_ [µM]	v_max_ [µmol/mg/min]
Idebenone	27	41.9	30	53.4	38	97.4
CoQ_1_	31	115.5	36	172.2	47	128.0
CoQ_10_	- †	- †	- †	- †	- †	- †
QS-10	8	20.5	13	23.3	5	29.6

*For NQO2 enzymatic assays 1-(3-sulfonatopropyl)-3-carbamoyl-1,4-dihydropyrimidine (NRH-derivative) was used as electron donor as described [36];

† No enzymatic activity above background could be detected for CoQ_10_, thus, steady-state kinetics could not be calculated.

NQO2, although much less studied, is reported to possess similar oxidoreductase activity with some differences in substrate specificities [35]. Despite similar cDNA and amino acid sequences of NQO1 and NQO2, NQO2 has different co-factor requirements (3). We were unable to demonstrate NQO2-dependent reduction of quinones using either NADH or NADPH as electron donor (data not shown), and used 1-(3-sulfonatopropyl)-3-carbamoyl-1,4-dihydropyrimidine, a synthetic analog of NRH [36], as electron donor instead. For all four quinones, we found similar results with regards to the K_m_ and v_max_ for NQO2-dependent reduction compared to the data generated with NQO1 (Table 1, Figure S1D).

### Cellular reduction of short-chain quinones by NQO1

**Figure 2:**
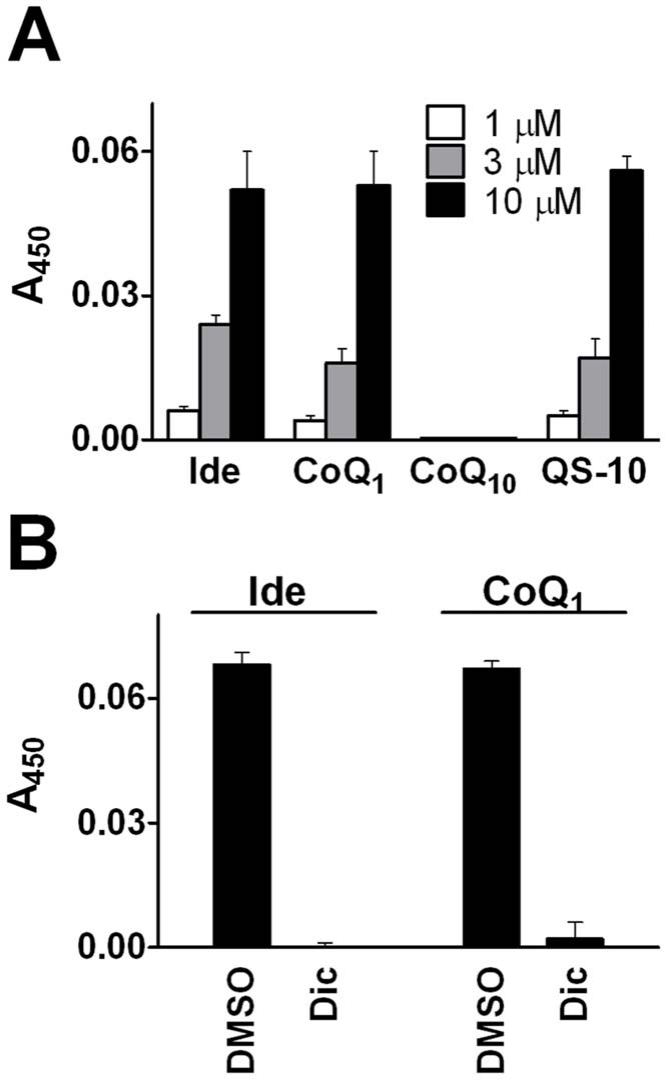
NQO1-dependent cellular reduction of quinones. (A) Dose-dependent cellular quinone reduction was measured as described by Tan *et al.* [37] in HepG2 cells. (B) Dicoumarol (Dic)-treatment (20 µM) efficiently blocked cellular quinone reduction in HepG2 cells. Bars represent mean +stdev of triplicates from one typical out of three independent experiments.

To confirm the *in vitro* reduction of short-chain quinones by NQO enzymes in cells, we employed an assay that measures the reduction-associated change in absorption of WST-1 to quantify NQO1-dependent reduction of quinones. A recent publication associated the quinone-dependent reduction of the tetrazolium dye WST-1 with NQO1 activity [37]. The authors demonstrated that WST-1 is converted only in the presence of functional NQO1, since inhibition of enzymatic activity by dicoumarol (Dic) abolished WST-1 reduction. Furthermore, the dye was potently reduced in cells expressing NQO1 but failed to change absorption in NQO1 deficient cells such as CHO cells. Indeed, using this assay, idebenone, CoQ_1_ and QS-10 were readily reduced by NQO1 in a dose-dependent manner in HepG2 cells; whereas for CoQ_10_ consistently no activity was detected (Figure 2A). Prior to differentiating between NQO1- and NQO2-dependent activities, it was essential to confirm the usefulness of the NQO1 inhibitor dicoumarol, with a reported IC_50_ for NQO1 of approximately 10 nM [38]. Consistent with previous reports [3], [35], our results showed that dicoumarol (20 µM) potently inhibited recombinant NQO1 activity (4% residual activity), while at the same time NQO2 activity was only inhibited by 14% (86% residual activity) (Figure S2). Therefore, co-incubation of HepG2 cells with quinones and dicoumarol for 120 minutes efficiently abolished the WST-1 signal (0% and 5% for idebenone and CoQ_1_, respectively) (Figure 2B). To rule out a cell line specific metabolism in HepG2 cells, comparable effects were also detected in primary fibroblasts and rat L6 myoblasts (Figures S3, S4). Reduction of substrates such as quinones by NQO1 uses NAD(P)H as electron donor. In agreement with previous reports using CoQ_1_ and β-lapachone [9], [39], idebenone reduced NADH levels in human lymphoblastoid cells in a dose-dependent manner. Using the NADH-dependent conversion of resazurin into the fluorescent resofurin product, idebenone reduced the fluorescence signal by 9% and 11% at (0.1 µM), 11% and 17% (1 µM) and 27% and 40% (10 µM) after 1- or 6-hours incubation, respectively (Figure S5A). Similarly, idebenone, CoQ_1_ and QS-10 decreased NADH levels after 3-hours incubation at a concentration of 10 µM quinone (69±4%, 62±0%, and 80±4% residual levels, respectively) (Figure S5B). In presence of dicoumarol (20 µM), the reduction of NADH was less prominent (80±5% with CoQ_1_) or was even prevented (105±15% and 92±6% by idebenone and QS-10, respectively). CoQ_10_ had no influence on NADH levels independent of a co-treatment with dicoumarol (100±1% without and 118±4% with dicoumarol).

### Effect of reduced quinones on rescue of rotenone-induced loss of ATP

**Figure 3:**
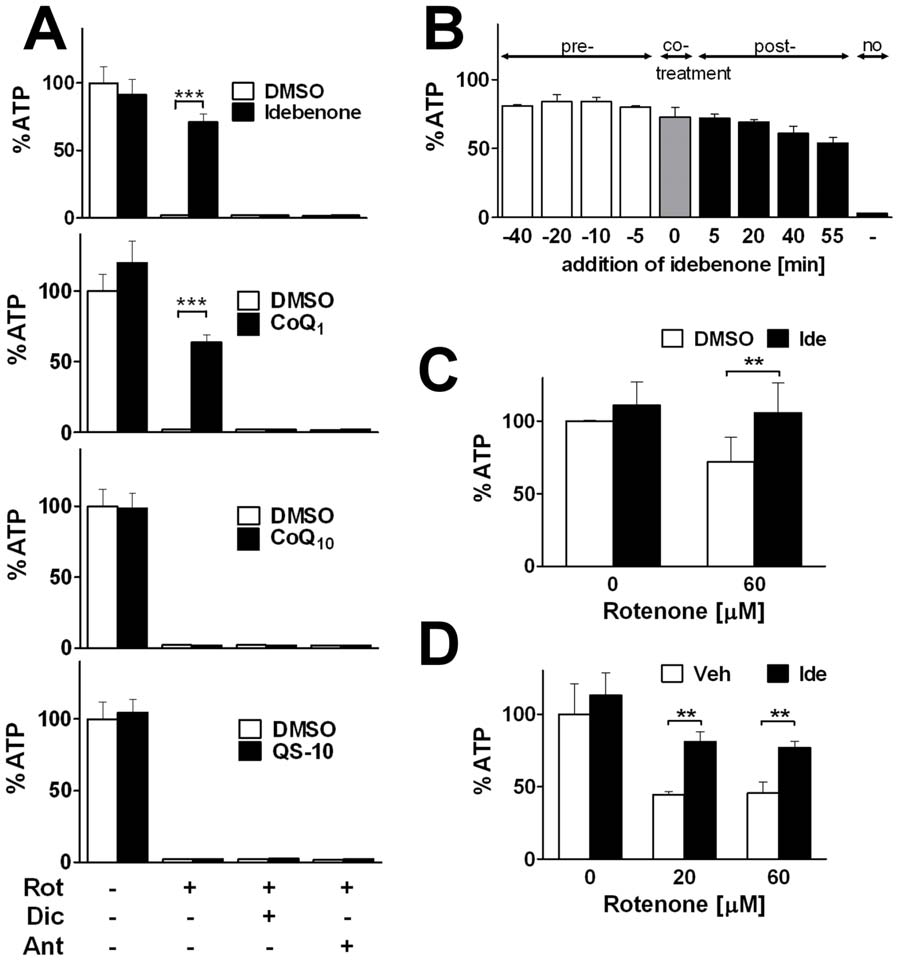
Idebenone and CoQ_1_ rescue ATP levels in complex I-repressed hepatocytes. (A) HepG2 cells were incubated with rotenone (Rot; 60 µM), dicoumarol (Dic; 20 µM) or antimycin A (Ant, 6 µM) in absence (empty bars) or presence (filled bars) of different quinones (5 µM idebenone, CoQ_1_, CoQ_10_ or QS-10) for 1 hour. ATP levels were normalized to protein and expressed as percentage of DMSO-treated cells in absence of rotenone. (B) HepG2 cells were incubated with 6 µM rotenone for 60 minutes, while 10 µM idebenone was pre-, co- or post-incubated regarding the time point of rotenone addition. ATP levels are expressed as percentage of untreated cells. Bars represent mean +stdev of triplicates of one typical out of two independent experiments. (C) Rescue of ATP levels of rotenone-treated (60 µM) primary mouse hepatocytes by acute idebenone treatment (5 µM) for 1 hour. Bars represent mean +stdev of six independent experiments. (D) Idebenone (400 mg/kg/day; p.o.) was administered to mice over 4 weeks and protection of ATP levels was maintained in rotenone-treated (20 and 60 µM for 1 hour) primary hepatocytes *ex vivo* without acute addition of idebenone. Bars represent mean +stdev of duplicates from each one idebenone- and sham-treated mouse. ATP levels were normalized to cell number and expressed as percentage of sham-treated hepatocytes in absence of rotenone. p**<0.01, p***<0.001.

It has been suggested that hydroquinones such as reduced CoQ_1_, despite their reduction in the cytosol, can donate electrons into the mitochondrial electron transport chain [8], [40], [41]. As a consequence, it was described that proton flux, membrane potential and ATP synthesis increased under conditions of impaired mitochondrial complex I function. We therefore determined the individual effectiveness of the related quinones for this cytosolic-mitochondrial electron transfer (Figure 3). In HepG2 cells, acute treatment of cells with the complex I inhibitor rotenone dramatically reduced ATP levels to 2% residual ATP while all four quinones left ATP levels unaffected (idebenone: 91±12%, CoQ_1_: 120±15%, CoQ_10_: 99±10%, and QS-10: 104±9% of control) (Figure 3A). However, under conditions of rotenone-induced ATP depletion (2±1% residual ATP), idebenone and CoQ_1_ partially restored ATP levels (71±6% or 64±6% of control levels, respectively) while CoQ_10_ and QS-10 were completely unable to restore ATP levels (2±1% for both quinones).

In the light of the results obtained in the cell-free system, we investigated to what extent the observed quinone-mediated rescue of ATP levels of complex I-inhibited cells was dependent on NQO1. In presence of rotenone, dicoumarol completely abolished the rescue of ATP levels normally induced by idebenone and CoQ_1_ (Figure 3A). Specifically, addition of 20 µM dicoumarol reduced ATP levels in presence of 60 µM rotenone from 71±6% and 64±6% residual ATP for idebenone and CoQ_1_, respectively, to 2% for both quinones (Figure 3A). Likewise, to address the question whether the quinone-dependent rescue of ATP levels is dependent on mitochondrial function we used the mitochondrial complex III inhibitor antimycin A (Ant). Analogous to the results obtained with dicoumarol, antimycin A prevented quinone dependent rescue of ATP levels (2% and 2±1% residual ATP with idebenone and CoQ_1_, respectively).

Recently, some evidence emerged that longer incubation periods of up to one week are required to detect some protective effects by CoQ_10_ [42]. Therefore, we investigated whether rescue of ATP levels, as demonstrated for acute exposure to idebenone and CoQ_1_, would be detectable after a 1-week treatment with CoQ_10_ (Figure S6). Rescue of ATP levels could not be detected for any quinone when administered only once at the beginning of a 1-week treatment. However, further addition of quinone simultaneously to the rotenone challenge after the 1-week treatment restored ATP levels in the case of idebenone and CoQ_1_, whereas under these conditions, CoQ_10_ again failed to protect ATP levels (Figure S6).

To test a possible time-dependency of the idebenone-mediated rescue of ATP levels, HepG2 cells were incubated with 6 µM rotenone for 60 minutes (Figure 3B). In addition, these cells were also treated with 10 µM idebenone for various incubation periods, either before or after the addition of rotenone (Figure 3B). Compared to rotenone-only treated cells (3±0% residual ATP), idebenone showed consistent protection of ATP levels in cells either pre-treated 40 minutes before the rotenone challenge (81±1% residual ATP) or cells simultaneous treated with rotenone and idebenone (73±7%) (Figure 3B). Interestingly, protection of ATP levels by idebenone was also evident, when it was added after the rotenone challenge. A 5-minute idebenone treatment still showed significant efficacy (54±4% residual ATP) in cells, which were already exposed to rotenone for 55 minutes (Figure 3B).

Similar results were observed in freshly isolated mouse hepatocytes. After isolation, hepatocytes were immediately treated with 60 µM rotenone in presence or absence of 5 µM idebenone. Again, idebenone protected cells from rotenone-induced ATP depletion (Figure 3C). Although, acute incubation of primary hepatocytes with rotenone did not lead to the same striking reduction of ATP levels compared to HepG2 cells (72±18% of control), idebenone fully restored ATP levels (106±21%) in this system. At the same time, in the absence of rotenone, idebenone did not alter ATP levels in these cells (111±16%).

This *ex vivo* activity of idebenone on ATP levels after rotenone-mediated impairment of complex I raised the question, whether this protective action could also be observed *in vivo*. Therefore, idebenone (400 mg/kg/day; p.o.) was administered to mice over a period of four weeks before hepatocytes were isolated and immediately treated with 20 µM or 60 µM rotenone for one hour as in previous experiments. In this experiment, however, idebenone was not freshly added to hepatocytes during this stress phase. Freshly isolated hepatocytes of idebenone-treated and sham- treated mice had similar basal ATP levels (113±16% and 100±21% respectively) (Figure 3D). Consistent with our *in vitro* and *ex vivo* data, rotenone led to a drop in ATP levels in hepatocytes of sham-treated animals (45±2% residual ATP levels at 20 µM rotenone, 46±8% at 60 µM rotenone). However, hepatocytes of idebenone-fed mice were significantly more resistant to rotenone challenge (81±7% residual ATP at 20 µM rotenone and 77±4% at 60 µM rotenone).

### ATP rescue is dependent on NQO1

**Figure 4:**
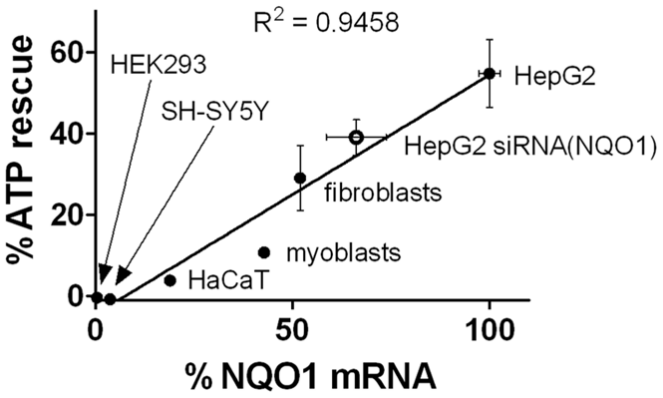
Rescue of ATP levels is dependent on NQO1. Correlation of ATP rescue and NQO1 mRNA expression in different human cell lines and primary cells. Percentage of ATP rescue by 10 µM idebenone in presence of 6 µM rotenone was defined as percentage of ATP levels in presence of rotenone and idebenone relative to the difference between ATP levels of DMSO- and rotenone-treated cells. mRNA levels were determined using qPCR and are relative to NQO1 expression in HepG2 cells. Results from HepG2 cells transduced with lentivirus encoding NQO1-specific shRNA are also included (open circle). Error bars represent standard deviation for both mRNA levels and ATP rescue (R^2^ = 0.9458).

Even though dicoumarol is reported to be a specific inhibitor of NQO1 [38], we wanted to rule out that other activities of dicoumarol, independent of NQO1, are responsible for the observed abolition of ATP rescue. Therefore, we investigated the ability of idebenone to rescue ATP levels after rotenone-challenge in cell lines and primary cells with different NQO1 expression levels (Figure 4). To compare the different cell lines, NQO1 mRNA levels, determined by qPCR, were normalized to HepG2 cells which showed the highest expression levels (mRNA levels: 100±2.7%; ATP rescue: 54.7±83%). In comparison, human embryonic kidney cells (HEK293) with very low NQO1 mRNA levels (0.4±1.2%) consistently failed to rescue ATP levels (−0.4±0.1%). Similarly, human neuroblastoma cells (SH-SY5Y) showed low NQO1 expression (3.7±0.3%) as well as ATP rescue capacity (−0.8±0.3%). In cells expressing higher NQO1 mRNA levels, such as human keratinocyte cell line (HaCaT) (18.9±0.0%), human myoblasts (42.8±1.2%) or human fibroblasts (52.0±0.5%), ATP rescue was more prominent (3.8±0.4%, 10.7±0.8% or 29.0±7.9%, respectively). Furthermore, downregulation of NQO1 expression in HepG2 cells by shRNA reduced mRNA levels (from 100±2.7% to 66.2±7.6%) as well as the ability to rescue ATP levels in presence of rotenone (from 54.7±8.3% to 39.0±4.4%). The data for all human cell lines tested clearly showed a positive correlation (R^2^ = 0.9458) of ATP rescue and NQO1 expression (Figure 4).

### Effect of quinones on lactate production by MELAS cybrids

**Figure 5:**
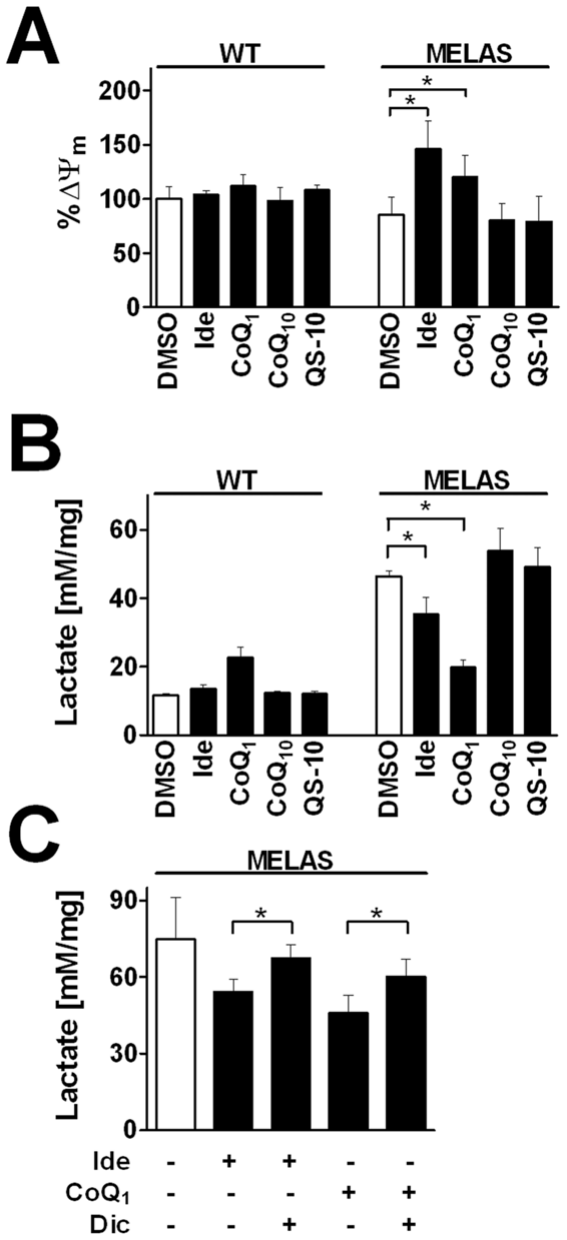
Effect of quinones on mitochondrial membrane potential and lactate production in MELAS cybrids. Cells were cultivated in galactose-containing media for 2 days in the presence or absence of quinones (10 µM). (A) Mitochondrial membrane potential (Δψ_m_) in wild-type (WT) and MELAS cybrids was measured using TMRM. Bars represent mean +stdev of 4 separate wells of a typical experiment as relative percentage compared to TMRM/protein in DMSO-treated WT cybrids (B) Lactate was measured in the supernatant and standardized to protein content. Data depict one typical experiment out of three and each data point represents mean + standard deviation of three individual wells. (C) Co-incubation with dicoumarol (10 µM) partially reverses the drop of lactate levels induced by idebenone or CoQ_1_. Extracellular lactate levels were standardized to protein content. Bars represent mean +stdev of 4 separate dishes within a typical experiment. p*<0.05, Student *t*-test.

Cells from *mitochondrial encephalomyopathy, lactic acidosis and stroke-like episodes* (MELAS) patients are characterized by impaired mitochondrial respiratory function. Mutations in mtDNA in these cells are generally associated with impaired function of mitochondrial complex I. As a consequence, low levels of ATP synthesis and excess production of lactate are described [43]. The observed excess lactate is largely a result of increased glycolysis to maintain sufficient energy levels under conditions of defective oxidative phosphorylation. The reason for producing lactate is to regenerate NAD^+^ levels which were utilized in the initial steps of glycolysis. Without sufficient NAD^+^, glycolysis cannot proceed. Since we showed quinone-dependent rescue of ATP levels under conditions of impaired mitochondrial complex I function (Figure 3) and NQO1-dependent metabolism is described to increase NAD^+^/NADH ratio [9], we investigated the role of quinones on cellular metabolism in cybrids harboring either wild type (WT) mitochondria or mitochondria from MELAS patients. If the effects of idebenone and CoQ_1_ in MELAS cybrids were comparable to those observed in healthy cells, it should strengthen mitochondrial respiration and, as a result, increase mitochondrial membrane potential (Δψ_m_). Although neither quinone changed Δψ_m_ in WT cybrids after a 2-day treatment, in MELAS cells, which have a slightly lower Δψ_m_ (85.0±16.9%) compared to DMSO-treated WT, Δψ_m_ was substantially increased after treatment with idebenone (145.8±26.2%) and CoQ_1_ (120.0±19.9%) (Figure 5A). Under these conditions, CoQ_10_ and QS-10 did not influence Δψ_m_ (80.3±15.2% and 78.8±23.9%). Unlike the situation where acute short-term incubation with quinones increased ATP levels after rotenone challenge (Figures 3,4), MELAS cells did not show increased ATP levels after 48-hour treatment (Figure S7). However, upon treatment with quinones for 48 hours, only idebenone and CoQ_1_ significantly reduced lactate levels by 24% and 57%, respectively (Figure 5B), which was partially reversed by addition of the NQO1-inhibitor dicoumarol (Figure 5C).

### Toxicological assessment of quinones

**Figure 6:**
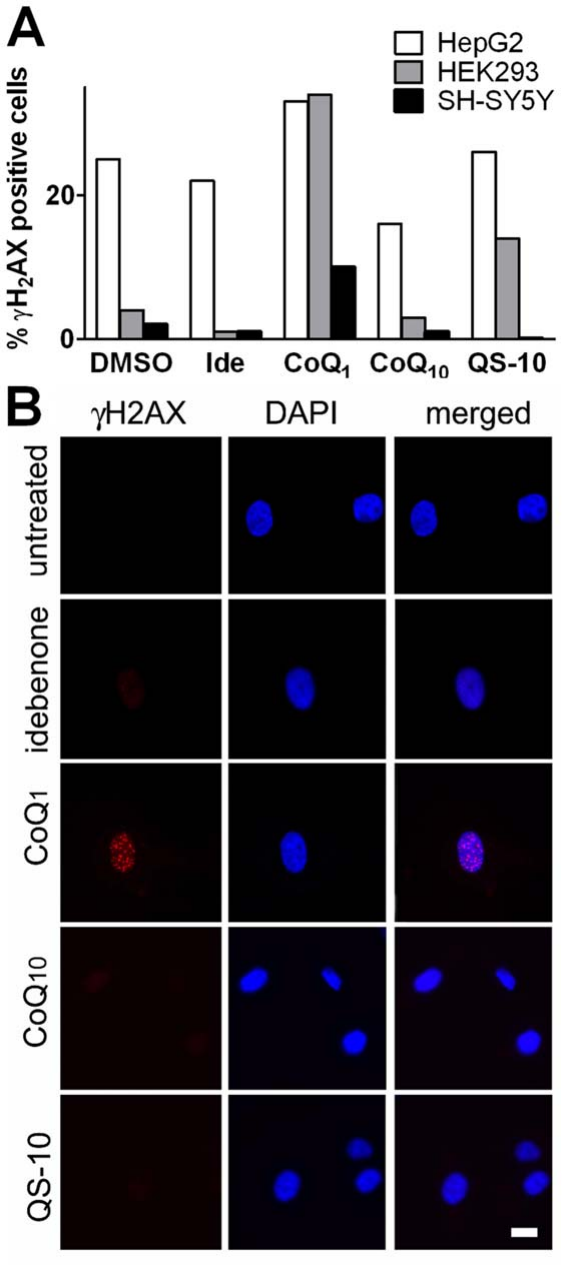
Genotoxic assessment of quinones. Induction of DNA damage by CoQ_1_. (A) HepG2, HEK293 and SH-SY5Y cells were cultured under culture conditions in presence of quinones (10 µM) for 24 hours before cells were fixed and stained against the DNA damage marker γH2AX. More than thousand cells per condition were counted manually for each condition and γH2AX-positive cells were expressed as percentage of the total number of cells counted. (B) Human primary fibroblasts were incubated for 72 hours with quinones (10 µM) under ambient conditions before cells were fixed and stained against the DNA damage marker γH2AX (red). DAPI dye was used as nuclear counterstain (blue). Scale bar: 10 µm.

Previous studies reported that idebenone and CoQ_1_ inhibit mitochondrial complex I function [11], [25], [29]–[31]. Based on results with other complex I inhibitors such as rotenone, it was suggested that some short-chain quinones could possess cytotoxic potential. Since a pro-oxidative function for some short-chain quinones was discussed [29], [31], we investigated the effects of the quinones tested in this study on cellular DNA damage in different cell lines. After 24-hour incubation with 10 µM quinones in normal medium, only CoQ_1_ showed a marked increase in γH2AX-positive cells (Figure 6A). This effect was most prominent in HEK293 cells (34% positive cells compared to 4% in sham-treated cells), but also in SH-SY5Y (10% compared to 2%), and amounted to only a slight increase in γH2AX-positive nuclei in HepG2 cells (33% compared to 25%). We extended this study also to primary cells. After 72-hour incubation of primary human fibroblasts with quinones (10 µM), only cells treated with CoQ_1_ were positive for the nuclear DNA damage marker γH2AX, while for all other quinones, including idebenone, no increase above basal levels could be detected (Figure 6B).

## Discussion

Quinones that are analogous to CoQ_10_ in the substitution pattern of their quinone moiety have often been proposed to share its biological activity. Just recently, Villalba et al. [44] suggested idebenone to be a good substitute for CoQ_10_ in different diseases. However, such predictions are questionable, since structural variances entail different chemical and physicochemical properties. Here, we have described that short-chain quinones are excellent substrates for reduction by NQO1 and NQO2, which is generally in agreement with previous reports [8], [40]. For instance, the obtained v_max_ for CoQ_1_ reduction by NQO1 is within the same range as reported by Beyer et al. [41]. In accordance to CoQ_1_, we have demonstrated here that idebenone and QS-10, an early metabolite of idebenone [32], are good substrates for NQO1. Strikingly, we did not detect any NQO1 or NQO2 activities above background when the lipophilic CoQ_10_ was used as electron acceptor, despite testing different formulations of CoQ_10_ such as liposomes. These results mirror a report by Siegel *et al.* [45], who described CoQ_10_ reduction by NQO1 with a v_max_ 3 orders of magnitute lower than the v_max_ we observed for CoQ_1_. Further publications reporting reduction of CoQ_10_ by NQO1 [reviewed in 46] display similar low velocities, which fall below the detection level of our system. These differences between the long-chain CoQ_10_ and its short-chain analogs were also observed in cells. The selective reduction of different quinones by NQO1, which was observed by us and others in different cellular systems, supports the idea of a general mechanism that is not cell type-dependent.

The relevance of NQO1-mediated reduction of short-chain quinones such as idebenone and CoQ_1_ lies in the fact that some hydroquinones can shuttle into the mitochondria and participate in mitochondrial electron transport. We were therefore interested if this phenomenon could restore energy levels under conditions of complex I deficiency. In this study, we observed a beneficial effect of idebenone and CoQ_1_ on cellular energy levels under conditions of acute inhibition of mitochondrial complex I by rotenone. Our results for CoQ_1_ are consistent with previously published data [8]. In this study, we demonstrate that idebenone also rescues ATP levels after acute complex I inhibition and that this action is dependent on both NQO1 and mitochondrial complex III. The dependency of NQO1 is not only shown by dicoumarol-mediated inhibition of enzymatic activity, but NQO1 expression in different cell lines and primary cells correlates well with the capacity to rescue ATP levels after rotenone challenge. In addition, partial silencing of NQO1 by RNAi reduces the idebenone mediated ATP rescue.

**Figure 7:**
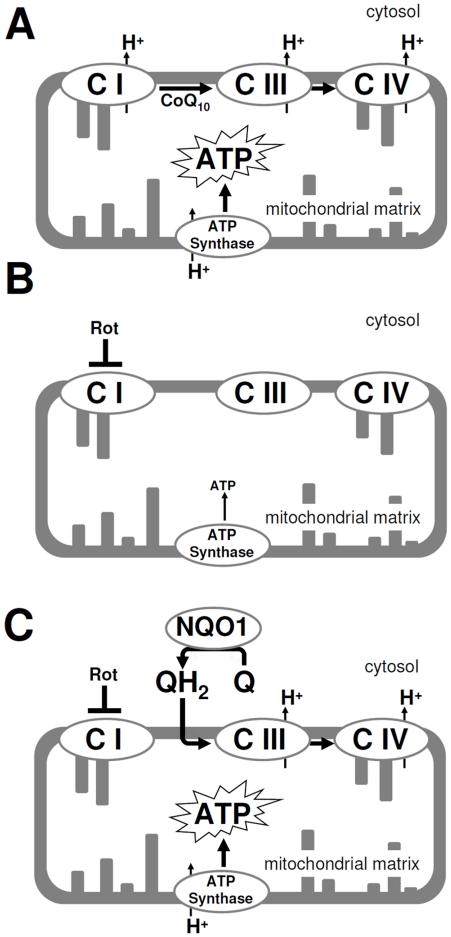
Schematic representation of NQO1-dependent cytosolic-mitochondrial electron shuttling. (A) During oxidative phosphorylation under normal conditions, CoQ_10_ transports electrons from complex I (CI) to complex III (CIII) and cytochrome *c,* reduced by complex III, transfers them to complex IV. As a consequence of this electron propagation, all three complexes translocate protons (H^+^) across the mitochondrial membrane, thus generating a proton gradient. ATP synthase utilizes the energy stored in this electro-chemical gradient to generate ATP. (B) Upon rotenone-induced (Rot) inhibition of complex I, ATP levels decrease dramatically (see also results of Fig. 3). (C) Some short-chain quinones (Q) such as idebenone or CoQ_1_ can bypass complex I inhibition via a cytosolic-mitochondrial shuttling of electrons. Upon reduction by cytosolic NQO1 (QH_2_), these quinones can feed electrons into the mitochondrial respiratory chain in a complex III-dependent manner, thereby restoring ATP production.

Both cytosolic and mitochondrial events are required for the quinone-dependent circumvention of complex I blockage of mitochondrial electron transport. Under normal conditions, mitochondrial complex I transfers two electrons from mitochondrial NADH to CoQ_10_ in the mitochondrial membrane which then passes the electrons on to cytochrome *c* in complex III. In contrast, the mechanism possibly used by idebenone and CoQ_1_ starts with the reduction of the quinone by NQO1 in the cytosol (Figure 7). Thereby, cytosolic NAD(P)H acts as the electron donor and substitutes for mitochondrial NADH as carrier of energy. The hydroquinone then enters the mitochondria to donate its electrons to complex III. Experiments by Degli Esposti et al. [29] revealed that reduced idebenone is a good substrate for complex III and can potently lead to reduction of cytochrome *c*. Since the hydroquinone is oxidized back into its quinone form by this reaction, a new cycle can be triggered resulting in a quinone-driven electron shuttle from cytosolic NAD(P)H to mitochondrial cytochrome *c*. Not only is this the first time that this mode of action has been described for the clinically used short-chain quinone idebenone, we have also provided evidence by treating animals with idebenone that this mechanism can operate *in vivo*.

Interestingly, when complex I was inhibited by rotenone for about one hour, a short, additional 5-minute incubation period with idebenone was still able to protect ATP levels. This suggests that idebenone is quickly absorbed and reduced by cellular systems and that restoration of decreased ATP levels can occur extremely fast. These findings provide a rationale why idebenone can be protective in disorders associated with impaired complex I function but normal levels of CoQ_10_. The reason for the extremely poor reduction of CoQ_10_ by NQO enzymes most likely originates from compartmentalization of enzyme and substrate. While NQO1 and NQO2 are strictly cytosolic enzymes [1], [3], CoQ_10_ is extremely hydrophobic and under physiological conditions only found integrated into biological membranes [47]. Therefore, CoQ_10_ cannot participate in this cytosolic-mitochondrial shuttling of electrons. Consistently, even prolonged cellular exposure to CoQ_10_ for up to one week failed to trigger an ATP rescue when complex I was dysfunctional. On the other hand, the surprising lack of detectable activity of QS-10, one of the first metabolites of idebenone [32], on restoring ATP levels, despite being reduced efficiently in cells and in cell-free conditions, could lie in its polarity. QS-10 is significantly more hydrophilic than idebenone and CoQ_1_, as manifested in a smaller log D value (Figure 1). It is therefore less likely that reduced QS-10 can pass through the lipophilic mitochondrial membrane to donate the electrons to complex III. Thus, our findings imply several requirements for this form of cytosolic-mitochondrial respiration. Not only is it necessary for quinones to enter the cytoplasm and show efficient reduction by NQO1, these compounds must also be able to enter the mitochondria. Then, within the mitochondria, they must be able to interact with complex III of the respiratory chain and release electrons that contribute to the mitochondrial proton gradient which is necessary for ATP synthesis.

Cybrid cells harboring the A3243G MELAS mutation possess a dysfunctional complex I. Thus, in order to generate sufficient ATP, they have to depend on anaerobic glycolysis. The price for increased glycolysis is the excessive production of lactate. Consistent with previous work [43], our data demonstrate that MELAS cybrids show more than 4-fold increased levels of extracellular lactate. It is interesting to note in this context that the main function of lactate production from pyruvate is entirely focused on regenerating NAD^+^ that is needed as co-factor for the initial steps of glycolysis. Here we show that quinone-mediated electron transfer from cytosolic NADH to mitochondrial complex III, as we described it for idebenone and CoQ_1_, is associated with increased mitochondrial membrane potential and an NQO1-dependent reduced lactate production of MELAS cybrids. The observation that at the same time both quinones were unable to increase ATP levels in cybrid cells suggests that MELAS cells are switching their metabolism from anaerobic glycolysis to mitochondrial respiration in order to generate the same levels of ATP. Since excess lactate production is considered to be one of the main pathological events in MELAS, this switch could be sufficient to alleviate some of the problems associated with the disease. Although we can not rule out a contribution of NQO1-quinone-dependent NAD^+^ production in the reduction of lactate levels observed in MELAS cells, we hypothesize that the mode of action lies predominantly in a quinone-dependent increase in mitochondrial activity.

Idebenone and CoQ_1_ have both been described to inhibit complex I [11], [25], [29]–[31]. As consequence of complex I inhibition, both quinones were suggested to also act as pro-oxidants under certain conditions [29], [31]. However, our results demonstrate that only CoQ_1_ but not idebenone triggered substantial DNA damage in different cell types. This clearly indicates that, despite sharing the protective activity against acute rotenone toxicity, idebenone does not cause DNA damage compared to CoQ_1_ after long-term administration. Because of these serious cytotoxic effects of CoQ_1_, we strongly caution against the use of CoQ_1_ in a clinical indication. Of the four quinones tested in this study, only idebenone met all requirements for cytosolic-mitochondrial redox cycling without evoking adverse effects (Table 2). Our findings also highlight the influence of modifications to the alkylic tail of short-chain quinones on their biological activity.

**Table 2:** Summary of the quinone characteristics.

	NQO substrate	Complex III substrate (reported)	Increased Δψ_m_	ATP rescue (in presence of rotenone)	Decrease of lactate (in MELAS)	Absence of toxicity
Idebenone	**+**	**+** *	**+**	**+**	**+**	**+**
CoQ_1_	**+**	**+** *	**+**	**+**	**+**	**-**
CoQ_10_	**-**	**+^†^**	**-**	**-**	**-**	**+**
QS-10	**+**	**-**	**-**	**-**	**-**	**+**

The abilities of the individual quinones to meet the requirements of cytosolic-mitochondrial shuttling and consequences thereof, as well as caveats for clinical administration, are listed.

*Idebenone and CoQ_1_ [29], as well as **†**CoQ_10_ [47], have been reported to be complex III substrates.

In summary, our data show that short-chain quinones possess entirely different activities compared to the lipophilic CoQ_10_ which suggests that they cannot substitute for each other. Some short-chain quinones such as idebenone, upon reduction by NQO1, generate a cytosolic-mitochondrial electron shuttle that can increase cellular energy levels, which can be utilized under conditions of impaired mitochondrial function. This mode of action appears promising for disorders characterized by complex I deficiencies such as MELAS, Leber's hereditary optic neuropathy (LHON) and Leigh's syndrome. However, without further testing for additional features of short-chain quinones, such as possible toxic liabilities, as shown here for CoQ_1_, extreme caution has to be exerted with regards to their therapeutic usefulness.

## Materials and Methods

### Ethical Statement

All animal experiments were approved by the governmental authorities (Kantonales Verterinäramt Basel-Land, Switzerland; permit number BL404) and were in accordance with international guidelines.

### Reagents and Chemicals

All chemical reagents, if not otherwise stated, were purchased from Sigma (Sigma-Aldrich, Buchs, Switzerland). All cell culture media and solutions, if not otherwise stated, were purchased from Omnilab (Zurich, Switzerland). Idebenone and QS-10 were synthesized in-house and were solid with purity ≥95% as determined by NMR and LCMS. For all assays described, compounds were dissolved at 10 mM (stock solution) in 100% DMSO (Acros Organics, Belgium).

### Cell culture and animal husbandry

Primary human fibroblasts (GM08402, Coriell, Camden NJ, USA), human neuroblastoma cell line SH-SY5Y (330154, Cell Line Services, Eppelheim, Germany), spontaneously transformed human keratinocyte cell line HaCaT (330493, CLS, Eppelheim, Germany), human embryonic kidney cell line HEK293 (CRL-1573, ATCC, Molsheim, France), rat L6 myoblasts (CRL-1458, ATCC, Molsheim, France), and human hepatic cell line HepG2 (330198, CLS, Eppelheim, Germany) cells were cultured under ambient conditions (37°C, 5% CO_2_, 90% humidity) in DMEM, 10% fetal bovine serum (FBS), Penicillin-Streptomycin-Glutamine. Lymphoblastoid cells (GM15851, Coriell) were cultured in RPMI 1640 under conditions as described above. Primary human myoblasts (from biopsy of healthy, 14-year old male, AFM, Evry, France) were cultured in MEM EBS supplemented with 25% M-199 EBS, 10% Hyclone FCS, 10 µg/ml insulin, 100 ng/ml EGF, 100 ng/ml FGF and Penicillin-Streptomycin-Glutamine under conditions described above. Wild-type (RN236, WT, homoplastic) and MELAS (RN164, A3243G homoplastic) cybrid cells [43] were cultured in DMEM, 7% FBS, Penicillin-Streptomycin-Glutamine and 50 µg/ml uridine. If not otherwise stated all animals were held under standard laboratory conditions (12 hours light per day, 22±2°C, 40–60% humidity) with food and water available ad libitum.

### NQO1 and NQO2 Activity

Recombinant NQO1 and NQO2 (Sigma, Buchs, Switzerland) activity in presence of different quinones was measured essentially according to a modified protocol by Ernster [48]. Reactions were performed in 1-ml disposable cuvettes at room temperature in reaction buffer (25 mM Tris-HCl pH 7.4, 0.7 mg/ml bovine serum albumin (BSA), 1 µg/ml enzyme, 10 µM quinone). The reaction was started by addition of NAD(P)H (for NQO1) or 1-(3-sulfonatopropyl)-3-carbamoyl-1,4-dihydropyrimidine (a NRH-derivate, for NQO2) [36]. Enzyme activity was measured as decrease of A_340_ for NAD(P)H and A_355_ for the NRH-derivate, respectively, during 30 seconds in a spectrophotometer (Ultrospec® 3000, Amersham Pharmaceutical Biotech, Little Chalfont, UK). All assays were performed in triplicate. Electron donor concentrations at start of linear phase of the decrease of absorbance were calculated using the absorbance coefficient (ε_NADH_ = 6300 M^−1^ cm^−1^; ε_NADPH_ = 6200 M^−1^ cm^−1^; ε_NRH-derivate_ = 4480 M^−1^ cm^−1^). Reduction rates per mg enzyme were calculated during the linear phase of the reduction. Since NQO1 possesses a single quinone-binding site [49], steady-state kinetic constants were calculated using the Michaelis-Menten equation combined with Hanes-Woolf plot because of its independence towards variability at high substrate levels. To determine the dicoumarol sensitivity of enzymes, reactions were performed in triplicate in the presence or absence of 20 µM dicoumarol in reaction buffer (25 mM Tris-HCl pH 7.4, 0.7 mg/ml BSA, and 1 µg/ml enzyme) containing 50 µM CoQ_1_ and started with 100 µM NADH or 1-(3-sulfonatopropyl)-3-carbamoyl-1,4-dihydropyrimidine, respectively. Electron donor consumption rate was calculated as described above and expressed as percentage of the rate in the absence of dicoumarol. For complexing quinones with serum, powdered quinones were dissolved in heat-inactivated FBS by vortexing for one minute. Alternatively, quinones were formulated in liposomes as described [33], [34]. Briefly, L-α-phosphatidylcholine and quinone were dissolved in PBS at a final concentration of 25 mg/ml lipid in a molar drug/lipid fraction of 0.05 (final quinone concentration: 1.6 mM). The mixture was then subjected to five repetitive freeze-thaw cycles.

### WST-1 assay for measuring NQO1-dependent quinone reduction in cells

WST-1 absorbance was determined as described previously [37]. Briefly, 96-well plates (Greiner, Frickenhausen, Germany) were seeded with 10^4^ HepG2 cells per well in DMEM with 2% FBS and 0.3 g/l glucose on the day before the WST-1 experiment. Inhibitors were preincubated for one hour using the following concentrations: dicoumarol 20 µM; rotenone 6 µM; antimycin 6 µM. After the preincubation time, the medium was replaced by Hank's balanced salt solution (HBSS; Omnilab, Zurich, Switzerland) containing 450 µM WST-1 (Dojindo Laboratories, Kumamoto, Japan) with or without inhibitors. The reaction was started by the addition of the quinone. WST-1 reduction (A_450_) was followed over a period of 120 minutes.

### Isolation of hepatocytes

Hepatocytes were isolated from 6-week old female NMRI mice (Janvier, France) as described [8]. Briefly, animals were sacrificed by CO_2_ and immediately perfused with 50 ml perfusion buffer (10 mM HEPES pH 7.4, 140 mM NaCl, 5 mM KCl, 2.5 mM Na_2_HPO_4_, 6 mM glucose and 0.2 mM EGTA; 37°C). The liver was removed and minced in pre-warmed collagenase buffer (10 mM HEPES pH 7.4, 140 mM NaCl, 5 mM KCl, 2.5 mM Na_2_HPO_4_, 6 mM glucose, 0.2 mM CaCl_2_, 1.3 mM MgSO_4_ and 0.05% collagenase D (Roche Diagnostics AG, Switzerland)). After incubation for 30 minutes at 37°C, hepatocytes were dissociated using a 5-ml syringe. The homogenous solution was filtered through gauze and the viability of cells was assessed using trypan blue staining. Typical viability of isolated hepatocytes was about 90%. For *ex vivo* studies with long-term treated mice, five-week-old male C57BL/6J mice were purchased from Janvier (France). After one week acclimatization period in the facility, the animals were single-housed and received a daily dose of 400 mg/kg idebenone in the food. For this, idebenone was dissolved in 0.5% carboxymethyl-cellulose by overnight stirring at 4°C. A 1:1 (w/w) mixture thereof with a normal chow/sugar (9∶1 w/w ratio) mash was prepared. Portions which amounted of approximately 75% of the daily calorie intake were stored at −20°C and administered just before start of the dark period. The portions for control animals were prepared identically with the exception of omitted idebenone. Additionally, mice had access to ad libitum food. Hepatocytes were isolated and treated as described before.

### Quinone-dependent rescue of ATP levels

HepG2 cells were seeded at a density of 10^5^ cells per well in a 96-well plate and incubated for 24 hours in DMEM without glucose, 2% FBS and Penicillin-Streptomycin-Glutamine. Cells were treated with 10 µM quinones in presence or absence of rotenone (60 µM), dicoumarol (20 µM) and antimycin A (6 µM) for 60 minutes in DMEM without glucose. Subsequently, cells were lysed and ATP levels were determined. Immediately after isolation, 10^6^ hepatocytes were diluted in 1 ml Krebs-Hensleit buffer (12.5 mM HEPES pH 7.4, 120 mM NaCl, 5 mM KCl, 1 mM KH_2_PO_4_, 1.2 mM MgSO_4_, 3 mM CaCl_2_, 24 mM NaHCO_3_,) and treated with different concentrations of quinones and inhibitors for 60 minutes at 37°C before ATP levels were determined.

### Quantification of ATP

Cellular ATP levels were quantified using luminescence from the ATP-dependent enzymatic oxidation of luciferin by luciferase. Briefly, isolated and treated cells were lysed in a volume of 200 µl (4 mM EDTA, 0.2% Triton X-100) for five minutes. In 96-well plates, 100 µl of ATP measurement buffer (25 mM HEPES pH 7.25, 300 µM D-luciferin, 5 µg/ml firefly luciferase, 75 µM DTT, 6.25 mM MgCl_2_, 625 µM EDTA and 1 mg/ml BSA) was combined with 10 µl lysate to start the reaction. Luminescence was quantified immediately using a multimode plate reader (Tecan M1000, Tecan iControl 1.6 software; Tecan Austria GmbH, Grödig, Austria). ATP levels were standardized to cell number for isolated hepatocytes or protein levels using BCA assay (ThermoScientific, Rockford, IL, USA) for cultured cells. Changes were calculated as percentage relative to levels of DMSO-treated control cells. ATP rescue is defined as the percentage of quinone-induced increase in ATP levels in presence of rotenone relative to the ATP reduction by rotenone alone.

### Lentiviral knock-down of NQO1

To knock down NQO1 expression, HepG2 cells were seeded in 12-well plates at 30000 cells per well in normal growth medium for 24 hours. Medium was replaced by 180 µl growth medium and 20 µl stock solution containing 10^5^ infectious units (IFU) of lentivirus encoding shRNA against NQO1 (sc-37139-V, Santa Cruz, Santa Cruz CA, USA) for a 24-hour incubation. Cells were then immediately used for quantifying NQO1 gene expression using qPCR and for ATP rescue experiments.

### mRNA levels

RNA was extracted from cultured cells using the High Pure RNA Isolation kit (Roche, Switzerland) according to the manufacturer's recommendations. Synthesis of first-strand cDNA was conducted using High Fidelity Transcriptor cDNA Synthesis kit (Roche, Switzerland) and random hexamer primers in a total volume of 20 µl containing 5 µg RNA. Real-time PCR was performed with Sybrgreen Real-Time PCR Master Mix (Roche, Switzerland) in a LightCycler 480 mastercycler and results were analyzed with the corresponding software (version 1.5.0.39). Protocol parameters used: 5 minutes at 95°C followed by 40 cycles of 10 seconds at 95°C for denaturing, 10 seconds at 56°C for annealing, and 10 seconds at 72°C for extension. GAPDH was used as internal control. Target gene sequences were amplified with the following primer pairs: NQO1 (forward: 5′-CACACTCCAGCAGACGCCCG-3′, reverse: 5′-TGCCCAAGTGATGGCCCACAG-3′) and GAPDH (forward: 5′-GAAGGTGAAGGTCGGAGTC-3′, reverse: 5′-GAAGATGGTGATGGGATTTC-3′).

### Measurement of mitochondrial membrane potential

MELAS and WT cybrid cells were seeded in black 96-well plates at 7500 cells per well in normal growth medium (DMEM, 4.5 mg/ml glucose, 10% FBS, 50 µg/ml uridine, Penicillin-Streptomycin). After 24 hours, the medium was changed to challenge medium (DMEM, 2 mg/ml glucose, 10% FBS, 50 µg/ml uridine, 2.5 mg/ml galactose, 0.11 mg/ml pyruvate, Penicillin-Streptomycin) containing DMSO or 10 µM quinones. After 48 hours, 50 µl of DMEM without glucose containing 3 µM tetramethylrhodamine methyl ester perchlorate (TMRM; Sigma-Aldrich, Buchs, Switzerland) was added on top. After 15 min incubation, cells were washed with warm PBS and 50 µl PBS was used for measurement of TMRM fluorescence using a multimode plate reader (Ex.: 545 nm; Em.: 580 nm; Tecan M1000). Fluorescence, corresponding to mitochondrial membrane potential (Δψ_m_), was standardized to protein content of lysates.

### Determination of extracellular lactate

MELAS and WT cybrid cells were seeded at a density of 1.5*10^5^ cells per 3.5-cm diameter cell culture dish in normal growth medium. After 24 hours, the medium was changed to challenge medium containing either DMSO or compounds. After 48 hours, the medium was removed for lactate measurement and the cells were lysed in 500 µl lysis solution (4 mM EDTA, 0.2% NP-40, 0.2% Tween-20) for 10 minutes. In a 96-well plate, 90 µl of reaction buffer (10 mM KH_2_PO_4_ pH 7.8, 2 mM EDTA, 1 mg/ml BSA, 0.6 mM DCPIP, 0.5 mM PMS, 0.8 mM NAD^+^, 1.5 mM glutamate, 5 U/ml glutamate-pyruvate-transaminase, 12.5 U/ml lactate dehydrogenase) was mixed with 10 µl medium. After incubation at 30°C for 30 minutes, absorption at 600 nm was quantified using a multimode plate reader (Tecan M1000). A lactate standard curve was run in parallel. Finally, the lactate concentration in the medium was standardized to protein content of the lysate using BCA assay.

### Genotoxic assessment of quinones

HepG2 cells, HEK293 cells, SH-SY5Y neuroblastoma cells and human primary fibroblasts were seeded in 8-chamber slides (Ibidi, Martinsried, Germany) under ambient conditions and treated for 24-hours with 10 µM quinones (72 hours for human primary fibroblasts). Cells were fixed using 4% PFA/PBS and stained against the DNA damage marker γH2AX (ab2893, Abcam, Cambridge, UK; 1∶1000 in TBST, 5% horse serum). DAPI dye was used as nuclear counter stain.

## Supporting Information

Figure S1 **Different quinones as substrates for NQO1 and NQO2.** Hanes-Woolf plots depict oxidation of (A) NADH or (B) NADPH by NQO1 in presence of different quinones as electron acceptors. Each data point represents the average of three independent measurements. (C) Effect of different quinone formulation in DMSO, liposomes (Lip) and fetal bovine serum (FBS) on metabolism by NQO1. Graph depicts electron donor oxidation rate expressed as percentage of control; mean +stdev of three independent measurements; p***<0.001, p**<0.01, two-tailed t-test. (D) Hanes-Woolf plot of NRH-derivate oxidation by NQO2 in presence of different quinone analogs. Each data point represents the average of three independent measurements.
(TIF)

Figure S2 **Specific inhibition of NQO1 by dicoumarol.** Dicoumarol (20 µM) selectively inhibited recombinant NQO1 activity (96% inhibition, filled bars) *in vitro*, whereas it reduced NQO2 activity by only 14% (empty bars). Graph depicts electron donor oxidation rate (%V_substrate oxidation_) expressed as percentage of control; mean +stdev of three independent measurements; p***<0.001, p**<0.01, two-tailed t-test.
(TIF)

Figure S3 **NQO1-dependent reduction of quinones in primary fibroblasts.** Dose-dependent cellular quinone reduction was measured as described [32] in human fibroblast cells. Bars represent mean +stdev of triplicates from one representative out of three independent experiments.
(TIF)

Figure S4 **NQO1-dependent reduction of quinones in rat L6 muscle cell line.** Dicoumarol (Dic)-treatment (20 µM) also efficiently blocked cellular quinone reduction in rat L6 cells. Bars represent mean +stdev of triplicates from one representative out of three independent experiments.
(TIF)

Figure S5 **NADH turnover in presence of quinones in human lymphoblastoid cells.** (A) Idebenone reduces NADH levels in a dose-dependent manner. (B) NADH levels are differently affected by treatment with idebenone, CoQ_1_, CoQ_10_ and QS-10 (10 µM) in absence (empty bars) or presence (filled bars) of dicoumarol (Dic; 20 µM). NADH content was measured using the NADH-dependent conversion of non-fluorescent resazurin into the fluorescent product resofurin. For cell culture experiments, 96-well black plates (Greiner, Frickhausen, Germany) were seeded with 10^5^ wild-type lymphoblastoid cells per well in 110 µl medium and compounds were added ranging from 0 to 10 µM. After one-day incubation at 37°C, cells were washed with PBS and resuspended in 110 µl phenol red-free RPMI. A volume of 10 µl cells was removed for protein determination. Resazurin was added to a final concentration of 4 µM and the cells were incubated at 37°C. Fluorescence change (Ex.: 544 nm, Em.: 590 nm) was measured at (A) 1 and 6 or (B) 3 hours. Wells containing medium and resazurin but no cells served to determine background fluorescence. Fluorescence signal was normalized to protein levels.
(TIF)

Figure S6 **ATP rescue after 1-week treatment.** HepG2 cells were seeded in to 96-well plates and treated for 1 week with 10 µM quinones under normal culture conditions. Medium was replaced by DMEM without glucose and cells were incubated for one hour in presence or absence of 6 µM rotenone. In addition, some of the wells were treated with fresh quinone (1 week + acute). After one hour, ATP levels were determined as described. Bars represent mean +stdev of one typical experiment.
(TIF)

Figure S7 **Effect of quinones on ATP levels in cybrid cells.** Cells were cultivated in galactose-containing challenge media for 2 days in the presence or absence of quinones (10 µM) and dicoumarol (10 µM). ATP levels were determined as described. Data depict one typical experiment out of three and each data point represents the mean +stdev of four individual dishes. p*<0.05, Student *t*-test.
(TIF)
