# Supplementary figures and images for: NQO1-Dependent Redox Cycling of Idebenone: Effects on Cellular Redox Potential and Energy Levels
Source: PLoS One. 2011 Mar 31;6(3):e17963. doi: 10.1371/journal.pone.0017963 (PMC3069029; doi:10.1371/journal.pone.0017963)

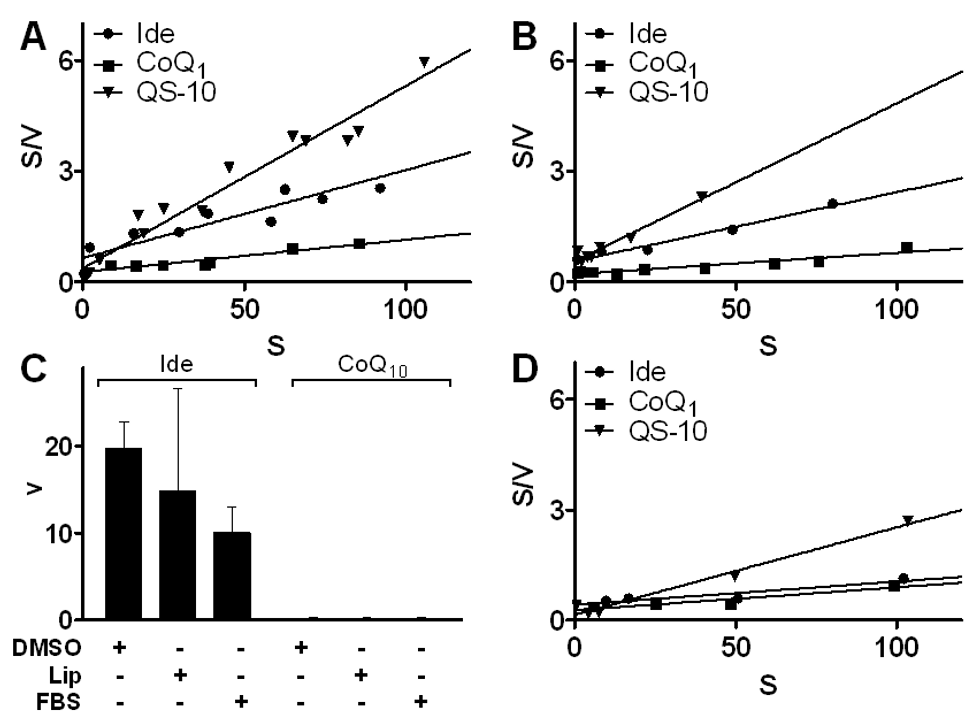

Supplement: Figure S1 — Different quinones as substrates for NQO1 and NQO2. Hanes-Woolf plots depict oxidation of (A) NADH or (B) NADPH by NQO1 in presence of different quinones as electron acceptors. Each data point represents the average of three independent measurements. (C) Effect of different quinone formulation in DMSO, liposomes (Lip) and fetal bovine serum (FBS) on metabolism by NQO1. Graph depicts electron donor oxidation rate expressed as percentage of control; mean +stdev of three independent measurements; p***<0.001, p**<0.01, two-tailed t-test. (D) Hanes-Woolf plot of NRH-derivate oxidation by NQO2 in presence of different quinone analogs. Each data point represents the average of three independent measurements. (TIF) [file pone.0017963.s001.tif]

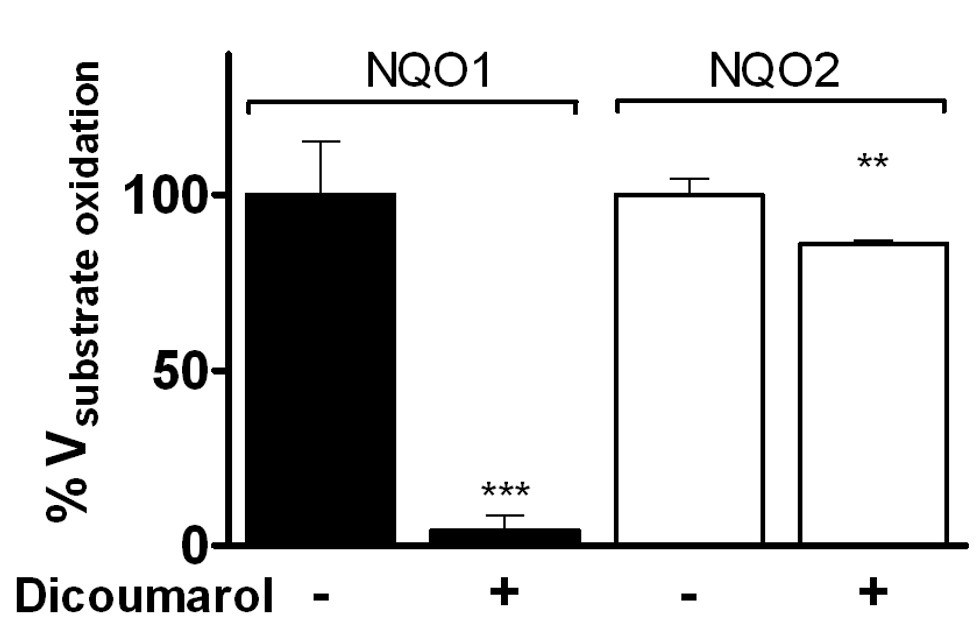

Supplement: Figure S2 — Specific inhibition of NQO1 by dicoumarol. Dicoumarol (20 µM) selectively inhibited recombinant NQO1 activity (96% inhibition, filled bars) in vitro, whereas it reduced NQO2 activity by only 14% (empty bars). Graph depicts electron donor oxidation rate (%Vsubstrate oxidation) expressed as percentage of control; mean +stdev of three independent measurements; p***<0.001, p**<0.01, two-tailed t-test. (TIF) [file pone.0017963.s002.tif]

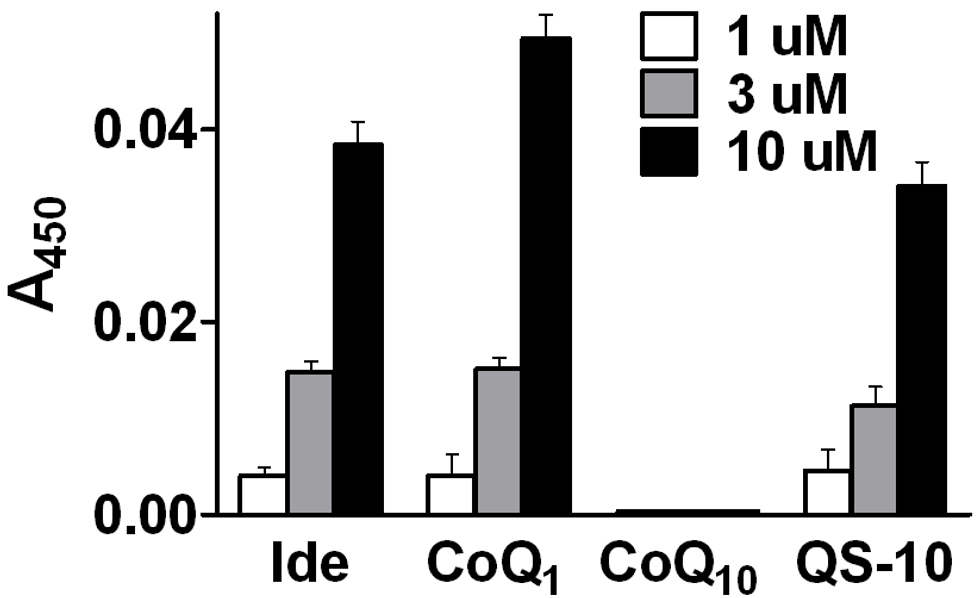

Supplement: Figure S3 — NQO1-dependent reduction of quinones in primary fibroblasts. Dose-dependent cellular quinone reduction was measured as described [32] in human fibroblast cells. Bars represent mean +stdev of triplicates from one representative out of three independent experiments. (TIF) [file pone.0017963.s003.tif]

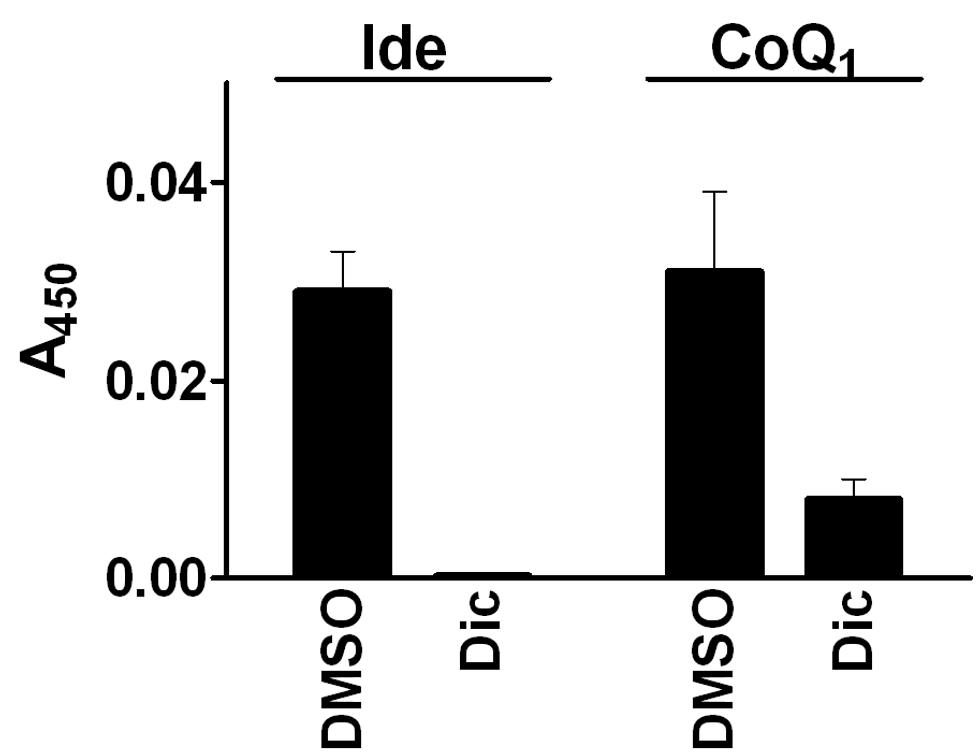

Supplement: Figure S4 — NQO1-dependent reduction of quinones in rat L6 muscle cell line. Dicoumarol (Dic)-treatment (20 µM) also efficiently blocked cellular quinone reduction in rat L6 cells. Bars represent mean +stdev of triplicates from one representative out of three independent experiments. (TIF) [file pone.0017963.s004.tif]

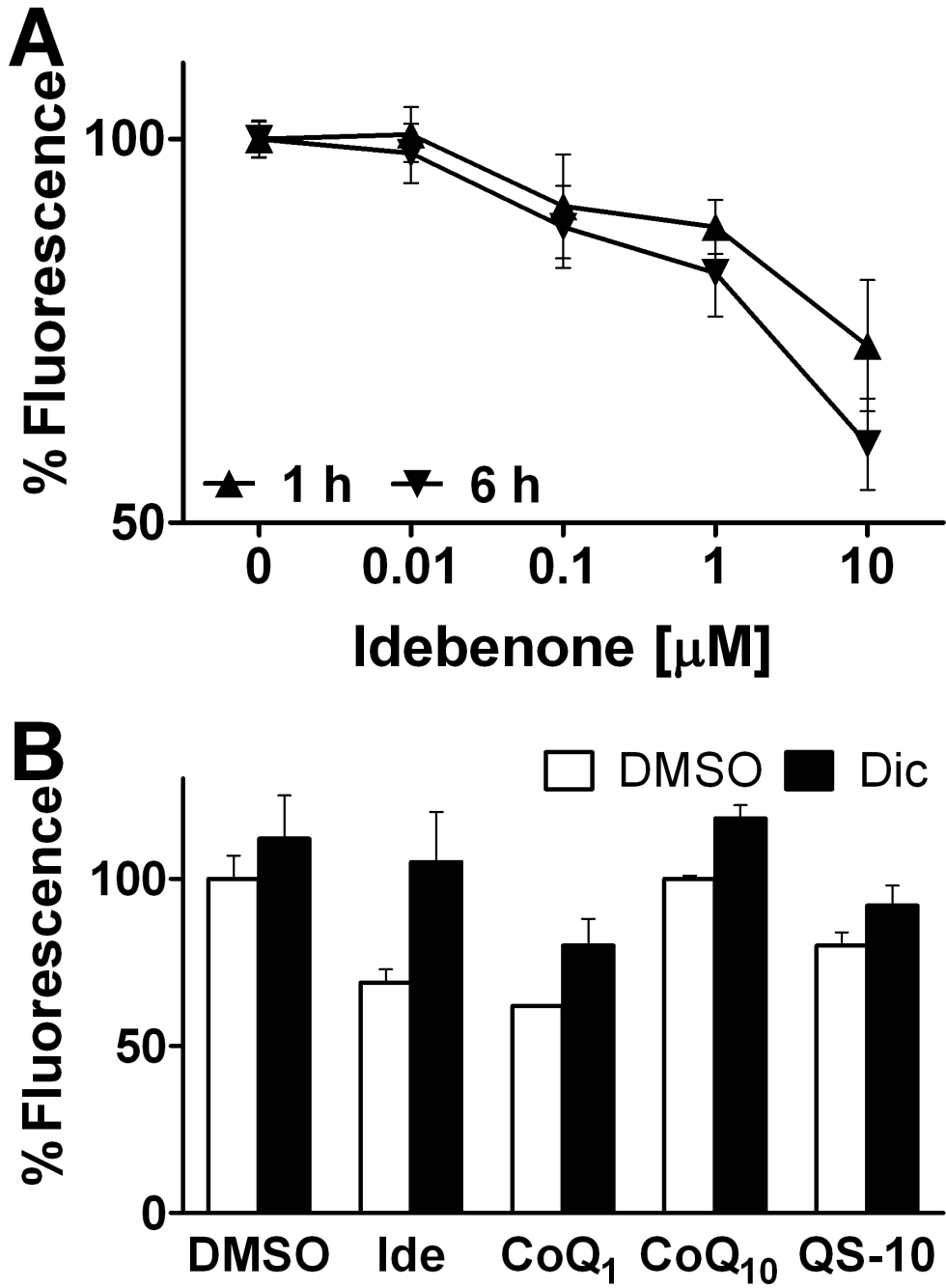

Supplement: Figure S5 — NADH turnover in presence of quinones in human lymphoblastoid cells. (A) Idebenone reduces NADH levels in a dose-dependent manner. (B) NADH levels are differently affected by treatment with idebenone, CoQ1, CoQ10 and QS-10 (10 µM) in absence (empty bars) or presence (filled bars) of dicoumarol (Dic; 20 µM). NADH content was measured using the NADH-dependent conversion of non-fluorescent resazurin into the fluorescent product resofurin. For cell culture experiments, 96-well black plates (Greiner, Frickhausen, Germany) were seeded with 105 wild-type lymphoblastoid cells per well in 110 µl medium and compounds were added ranging from 0 to 10 µM. After one-day incubation at 37°C, cells were washed with PBS and resuspended in 110 µl phenol red-free RPMI. A volume of 10 µl cells was removed for protein determination. Resazurin was added to a final concentration of 4 µM and the cells were incubated at 37°C. Fluorescence change (Ex.: 544 nm, Em.: 590 nm) was measured at (A) 1 and 6 or (B) 3 hours. Wells containing medium and resazurin but no cells served to determine background fluorescence. Fluorescence signal was normalized to protein levels. (TIF) [file pone.0017963.s005.tif]

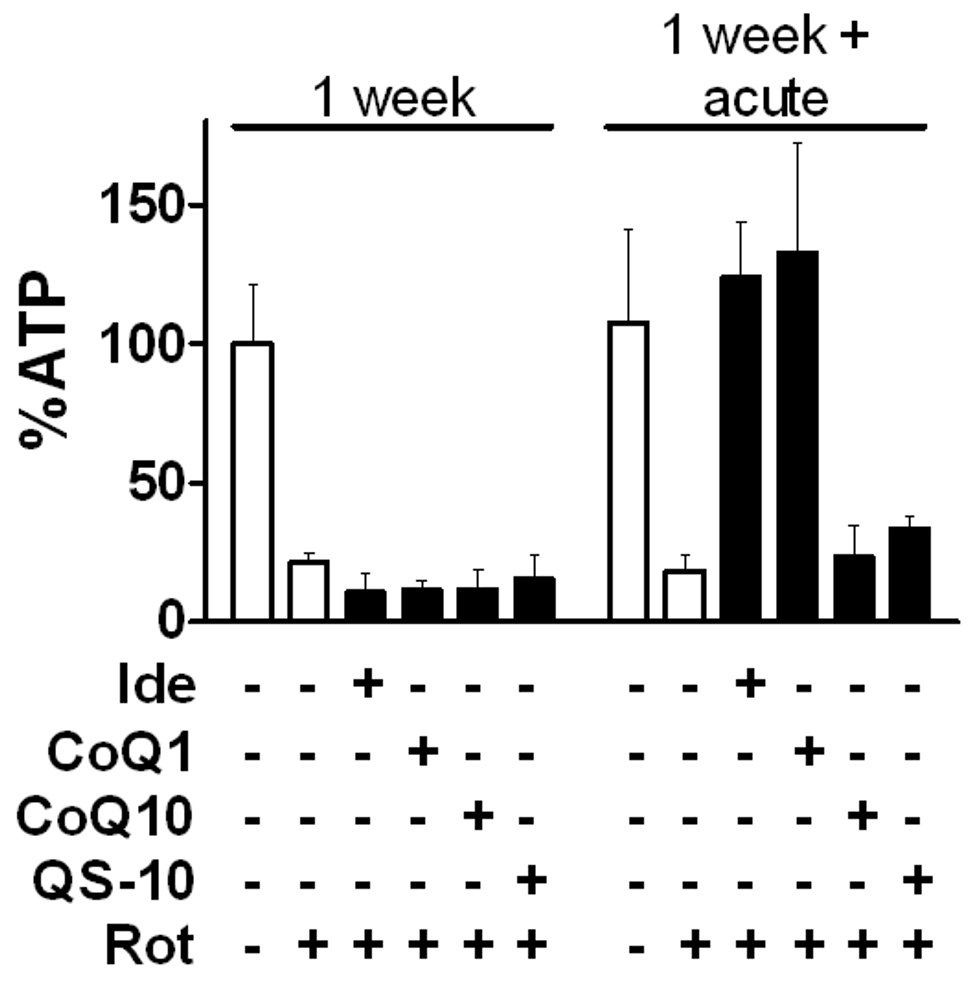

Supplement: Figure S6 — ATP rescue after 1-week treatment. HepG2 cells were seeded in to 96-well plates and treated for 1 week with 10 µM quinones under normal culture conditions. Medium was replaced by DMEM without glucose and cells were incubated for one hour in presence or absence of 6 µM rotenone. In addition, some of the wells were treated with fresh quinone (1 week + acute). After one hour, ATP levels were determined as described. Bars represent mean +stdev of one typical experiment. (TIF) [file pone.0017963.s006.tif]

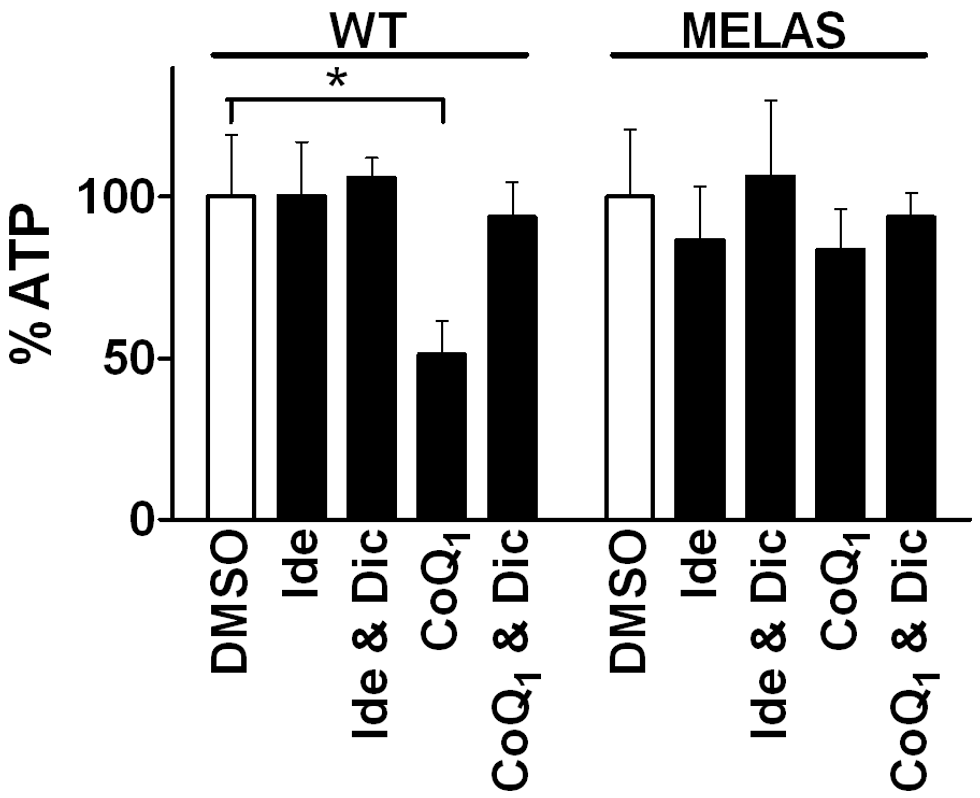

Supplement: Figure S7 — Effect of quinones on ATP levels in cybrid cells. Cells were cultivated in galactose-containing challenge media for 2 days in the presence or absence of quinones (10 µM) and dicoumarol (10 µM). ATP levels were determined as described. Data depict one typical experiment out of three and each data point represents the mean +stdev of four individual dishes. p*<0.05, Student t-test. (TIF) [file pone.0017963.s007.tif]
